# Autophagy Functions to Prevent Methylglyoxal-Induced Apoptosis in HK-2 Cells

**DOI:** 10.1155/2020/8340695

**Published:** 2020-06-04

**Authors:** So-Hyun Park, Hyun-Il Choi, Jiyun Ahn, Young-Jin Jang, Tae-Youl Ha, Hyo-Deok Seo, Yoon-Sook Kim, Dae-Hee Lee, Chang Hwa Jung

**Affiliations:** ^1^Division of Food Functionality Research, Korea Food Research Institute, Wanju-gun, Jeollabuk-do 55365, Republic of Korea; ^2^Department of Food Biotechnology, Korea University of Science and Technology, Wanju-gun, Jeollabuk-do 55365, Republic of Korea; ^3^Department of Marine Food Science and Technology, Gangneung-Wonju National University, Gangneung, Gangwon-do 25457, Republic of Korea

## Abstract

Methylglyoxal (MGO), a reactive carbonyl species, causes cellular damage and is closely related to kidney disease, particularly diabetic nephropathy. Although MGO has been reported to induce autophagy and apoptosis, the relationships between the two pathways are unclear. Here, we evaluated whether autophagy may be the underlying mechanism inhibiting MGO-induced apoptosis. MGO treatment induced concentration- and time-dependent apoptosis in HK-2 cells. Moreover, MGO upregulated the autophagy markers p62 and LC3-II. Apoptosis caused by MGO was increased in ATG5-knockdown cells compared to that in wild-type cells. In contrast, autophagy activation by 5-aminoimidazole-4-carboxamide ribonucleotide resulted in reduced apoptosis, suggesting that autophagy played a role in protecting against MGO-induced cell death. To examine the mechanisms through which autophagy occurred following MGO stimulation, we investigated changes in AKT/mammalian target of rapamycin (mTOR) signaling. Autophagy induction by MGO treatment was not related to AKT/mTOR signaling; however, it did involve autophagy-related gene expression promoted by AMP-activated protein kinase-mediated transcription factors, such as forkhead box 1. Overall, our findings indicate that MGO-induced cellular damage can be mitigated by autophagy, suggesting that autophagy may be a potential therapeutic target for diseases such as diabetic nephropathy.

## 1. Introduction

Methylglyoxal (MGO), a reactive carbonyl species, is formed in the glycolytic pathway following fragmentation of glyceraldehyde-3-phosphate and dihydroxyacetone phosphate [1]. MGO is a major precursor of advanced glycation end products and can induce intracellular damage by increasing reactive oxygen species levels and mitochondrial damage, leading to apoptosis [2–4]. According to previous studies, MGO is produced rapidly under hyperglycemic conditions in diabetic patients, and accumulated MGO is then involved in several diseases, such as cognitive dysfunction, cardiovascular diseases, and bone loss [5–10]. In particular, MGO contributes to the development of diabetic microvascular complications, such as nephropathy and retinopathy [11–13]. Although a recent study revealed that MGO is related to the early progression of diabetic nephropathy, the molecular mechanisms of the cytotoxic effects of MGO in kidney cells remain unclear [14].

Autophagy is an intracellular degradation mechanism that targets damaged organelles, lipids, and aggregated proteins [15]. Importantly, autophagy has critical roles in cell survival and cell death responses to intracellular stress. Indeed, previous studies have shown that autophagy alleviates cell death in a variety of stress environments, including starvation, oxidative stress, and DNA damage [16–18]. Additionally, autophagy inducers, such as 5-aminoimidazole-4-carboxamide ribonucleotide (AICAR) and rapamycin, also block various stress-induced cell death pathways, suggesting a correlation between MGO-induced autophagy and apoptosis [19, 20]. Autophagy activation in human brain microvascular epidermal cells protects against MGO-induced cell damage [21]. Luteolin, a type of phytochemical flavonoid, also inhibits cell death in MGO-treated cells by blocking mammalian target of rapamycin (mTOR) signaling [22]. However, the effects of autophagy in MGO-induced apoptosis and its associated mechanisms have not been fully elucidated in kidney cells.

Accordingly, in this study, we investigated whether autophagy had protective effects against MGO-induced apoptosis and examined the mechanisms through which autophagy was caused by MGO.

## 2. Materials and Methods

### 2.1. Cell Culture

Human kidney (HK-2) and SV40MES13 cells were purchased from the American Type Culture Collection (Manassas, VA, USA). HK-2 cells were cultured in Roswell Park Memorial Institute 1640 medium supplemented with 10% fetal bovine serum (FBS) and 1% penicillin-streptomycin (PS). SV40MES13 cells were cultured in a 3 : 1 mixture of DMEM/F-12 medium supplemented with 5% FBS, 14 mM of HEPES, and 1% PS at 37°C in an atmosphere containing 5% CO_2_.

### 2.2. Cell Viability

Cells were seeded in 96-well plates at a density of 1 × 10^4^ cells/well in 100 *μ*L complete medium and incubated for 24 h. The cells were treated with various concentrations (0–1 mM) of MGO with 100 *μ*L complete medium for 24 or 48 h. Following treatment, 20 *μ*L MTT solution (5 mg/mL in phosphate-buffered saline) was added to each well, and cells were incubated for an additional 2 h. Next, the medium was removed, and 200 *μ*L dimethyl sulfoxide was added to each well. The cell viability was determined by measuring absorbance at 540 nm using an Infinite M200 microplate reader (Tecan US, Inc., Morrisville, NC, USA).

### 2.3. Terminal Deoxynucleotidyl Transferase dUTP Nick-End Labeling (TUNEL) Assay

Apoptotic cells were detected using a DeadEnd Fluorometric TUNEL System kit (cat. no. G3250; Promega, Madison, WI, USA) according to the manufacturer's instructions. Apoptotic cells were imaged using a fluorescent microscope (Olympus IX71, Japan).

### 2.4. Annexin V and Dead Cell Assays

The apoptotic cell death ratio was determined using a Muse Annexin V and Dead Cell Assay Kit (cat. no. MCH100105; Merck Millipore, Billerica, MA, USA). Briefly, HK-2 cells were seeded into 6-well plates (2 × 10^5^ cells/well). After 24 h, cells were treated with 0.5 or 0.75 mM MGO for 24 h. After treatment, all cells were harvested and stained with annexin V solution for 20 min. Apoptotic cell death was measured using a MUSE cell analyzer (Merck Millipore).

### 2.5. Immunoblotting

Cellular proteins were harvested using cell lysis buffer (cat. no. 9803S; Cell Signaling Technology, Danvers, MA, USA) supplemented with protease and phosphatase inhibitor cocktails (Thermo Fisher Scientific, Waltham, MA, USA). The harvested cells were centrifuged at 13,000 rpm for 10 min at 4°C, and the supernatants were separated. The proteins were quantified using Bradford assays and separated by sodium dodecyl sulfate polyacrylamide gel electrophoresis. The separated proteins were transferred to polyvinylidene fluoride membranes. The membranes were then blocked with 5% skim milk for 1 h and incubated overnight at 4°C with the following primary antibodies: anti-phospho-AMP-activated protein kinase (AMPK; Thr172; cat. no. 2535; Cell Signaling Technology), anti-AMPK (cat. no. 2793; Cell Signaling Technology), anti-microtubule-associated protein 1A/1B light chain 3B (LC3B; cat. no. 2775; Cell Signaling Technology), anti-phospho-ribosomal protein S6 kinase *β*-1 (S6K1; Thr389; cat. no. 9234; Cell Signaling Technology), anti-S6K1 (cat. no. 9202; Cell Signaling Technology), anti-poly-ADP ribose polymerase (PARP; cat. no. 9542; Cell Signaling Technology), anti-p62/SQSTM-1 (cat. no. P0067; Sigma-Aldrich, St. Louis, MO, USA), anti-Atg5 (cat. no. NB110-53818; Novus Biologicals, CO, USA), anti-Caspase-3 (cat. no. 9662; Cell Signaling Technology), anti-glyceraldehyde 3-phosphate dehydrogenase (cat. no. sc-365062; Santa Cruz Biotechnology), anti-Cytochrome C (cat. no. sc-13156, Santa Cruz Biotechnology), and anti-*β*-actin (cat. no. sc-47778; Santa Cruz Biotechnology). The next day, the membranes were washed and incubated with secondary antibodies for 1 h at room temperature. Protein bands were visualized using G:BOX Chemi XX6 (Syngene Ltd., Frederick, MD, USA) with an enhanced chemiluminescence substrate. The intensities of the protein bands were measured using ImageJ software ver. 1.8 (National Institutes of Health, MD, USA).

### 2.6. Real-Time Quantitative Polymerase Chain Reaction (RT-qPCR)

RNA extraction and cDNA synthesis were performed using a RNeasy Mini Kit (Qiagen, Inc., Valencia, CA, USA) and a ReverTra Ace qPCR RT Kit (Toyobo Co., Ltd., Osaka, Japan) according to the manufacturer's instructions. qPCR was then performed using the SYBR Green Realtime PCR Master Mix (Toyobo Co., Ltd.) with a Viia 7 Real-Time PCR system (Thermo Fisher Scientific). Relative RNA levels were normalized according to the levels of actin mRNA. The specific primer sequences are listed in Table 1.

### 2.7. Immunofluorescence

HK-2 cells were seeded into 8-well chamber slides (2.5 × 10^4^ cells/well). After 24 h, cells were treated with MGO. Immunostaining was performed as previously described [23]. Briefly, cells were fixed with 4% formaldehyde for 15 min and permeabilized with 0.05% saponin for 30 min at room temperature. Cells were incubated with 1% bovine serum albumin blocking solution and reacted with anti-LC3B antibody (cat. no. 2775; Cell Signaling Technology) overnight at 4°C. The next day, cells were washed thrice and stained with rabbit secondary antibody (cat. no. 4412; Cell Signaling Technology). After 30 min, the cells were washed with PBS and incubated with DAPI solution (cat. no. 10236276001; Sigma-Aldrich) for 5 min. Microscopy was performed at 408 nm (4′6-diamidino-2-phenylindole) and 488 nm (fluorescein isothiocyanate) using an Eclipse Ti confocal fluorescent microscope (Nikon, Tokyo, Japan).

### 2.8. siRNA Transfection

siRNAs targeting Atg5 and AMPK*α* and nontargeted siRNA (siNC) were purchased from Dharmacon (CO, USA). HK-2 cells were seeded into 6-well plates (2 × 10^5^ cells/well) and incubated until at least 60% confluence was attained. The cells were transfected with siAtg5 or siAMPK or siNC using Lipofectamine RNAi Max (Thermo Fisher Scientific) according to the manufacturer's instructions. siRNA was added to Opti-MEM with a transfection reagent and incubated at room temperature for 20 min. The mixture was then added to each well, and the cells were incubated for an additional 24 h. After transfection, cells were harvested and used for subsequent experiments.

### 2.9. Statistical Analysis

All data are presented as means ± standard deviations. The significance of differences was evaluated by one-way analysis of variance, followed by Tukey's post hoc multiple comparison test using GraphPad Prism 7 software (GraphPad Software, San Diego, CA, USA). Differences with *p* values of less than 0.05 were considered significant.

## 3. Results

### 3.1. MGO Treatment Induced Apoptosis in HK-2 Cells

**Figure 1:**
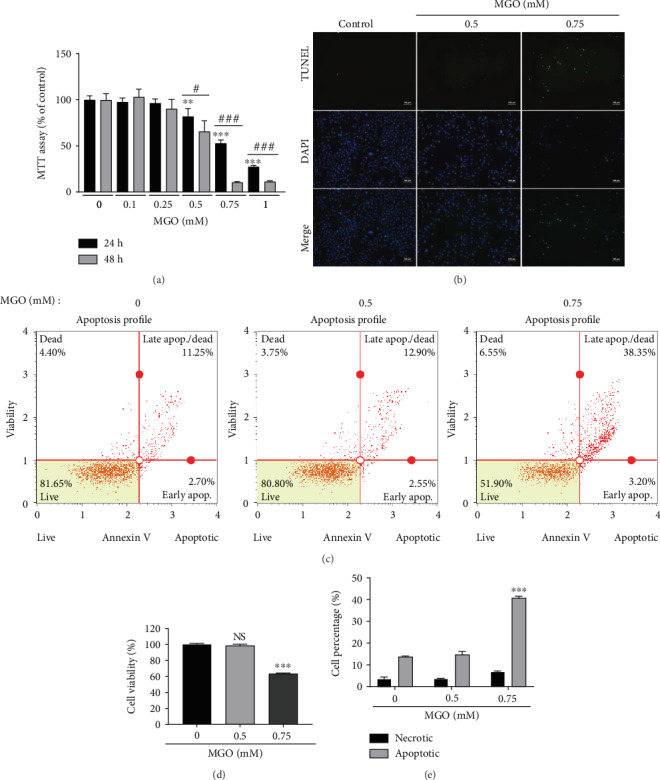
MGO treatment induces cell death in HK-2 cells. (a) HK-2 cells were treated with the indicated concentrations (0–1 mM) of MGO for 24 or 48 h, and MTT assays were conducted. (b) Representative image of apoptotic cells in HK-2 cells treated with MGO for 24 h detected by TUNEL assays (original magnification: 100x). (c–e) Representative flow cytometry analysis of HK-2 cells treated with MGO for 24 h and then stained with the Muse Annexin V and Dead Cell Kit. (c) Flow cytometry density plot of MGO-treated HK-2 cells. (d) Cell viability and (e) total apoptotic cell percentages following treatment with 0–0.75 mM MGO. Values are mean ± standard deviation. ^∗∗^*p* < 0.01 and ^∗∗∗^*p* < 0.001 versus the control (0) group; ^#^*p* < 0.05 and ^###^*p* < 0.001 versus the same concentration of MGO (*n* = 3).

MGO is known to induce cell death and suppress cell proliferation [24, 25]. Therefore, we first evaluated the appropriate concentration of MGO for reducing cell viability in HK-2 cells using MTT assays with various concentrations of MGO. The results showed that MGO decreased cell viability in a concentration- and time-dependent manner and significantly induced cell death following treatment with MGO for 24 h at a concentration above 0.5 mM (Figure 1(a), Figure S1(a)). We then confirmed the apoptosis-inducing concentration (0.75 mM) using TUNEL assays, showing increased formation of green dots representing DNA fragmentation (Figure 1(b)). Muse Annexin V and Dead Cell Assays showed that the proportion of apoptotic cells was significantly increased following treatment with 0.75 mM MGO; however, the increase in necrotic cell death was not significant (Figures 1(c)–1(e)). These results indicate that treatment with 0.75 mM MGO for 24 h in HK-2 cells induced apoptosis in approximately 40% of the cells.

### 3.2. MGO Treatment Induced Autophagy

**Figure 2:**
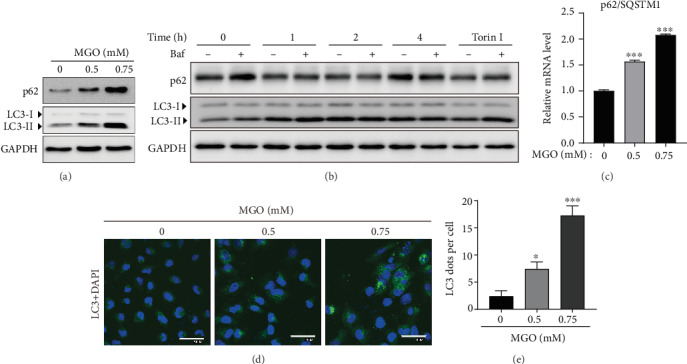
MGO treatment enhances autophagy. (a) Expression of autophagy-related proteins in HK-2 cells treated with MGO (0.5–0.75 mM) for 24 h. (b) The time-dependent protein expression levels of LC3 and p62 in HK-2 cells treated with MGO (0.75 mM) and torin 1 (positive control, 200 nM, 4 h) with or without Baf (50 nM, 1 h). (c) Transcriptional levels of p62/SQSTM-1 mRNA. (d) Representative immunofluorescence image of HK-2 cells treated with MGO for 24 h (green: LC3; blue: DAPI; original magnification: 600x). (e) LC3 puncta following treatment with MGO. Values are mean ± standard deviation. ^∗^*p* < 0.05 and ^∗∗∗^*p* < 0.001 versus vehicle (0) group.

According to recent reports, MGO increases autophagy in cells [25, 26]. Therefore, we next investigated whether autophagy occurs at apoptosis-inducing MGO concentrations. Autophagy protein markers, such as p62/SQSTM-1 and LC3-II, were increased in a concentration-dependent manner following treatment with MGO (Figure 2(a)). In general, when autophagy is activated, LC3-II protein expression is increased while the expression of p62/SQSTM-1 is decreased [27]. Alternatively, the inhibition of autophagy flux can induce the accumulation of p62/SQSTM-1. We, therefore, sought to evaluate autophagy flux using bafilomycin A1 (baf), an autophagosome-lysosome fusion inhibitor, and found that MGO significantly increased protein levels of LC3 over 1–4 h, and the accumulation of LC3 induced by baf was increased in MGO-treated cells compared with that in baf control cells, similarly to what was observed for torin 1 (autophagy activator, positive control) (Figure 2(b)). In addition, our data showed that MGO enhanced p62/SQSTM-1 mRNA (Figure 2(c)), suggesting that MGO activated autophagy flux, and the autophagy enhancement was associated with p62/SQSTM-1 mRNA expression. Moreover, the numbers of autophagosome puncta in MGO-treated cells was increased compared with that in the control group (Figures 2(d) and 2(e)). Collectively, our results suggested that MGO enhanced autophagy in HK-2 cells and increased the expression of autophagy-related gene.

### 3.3. Autophagy Had Protective Effects against MGO-Induced Apoptosis

**Figure 3:**
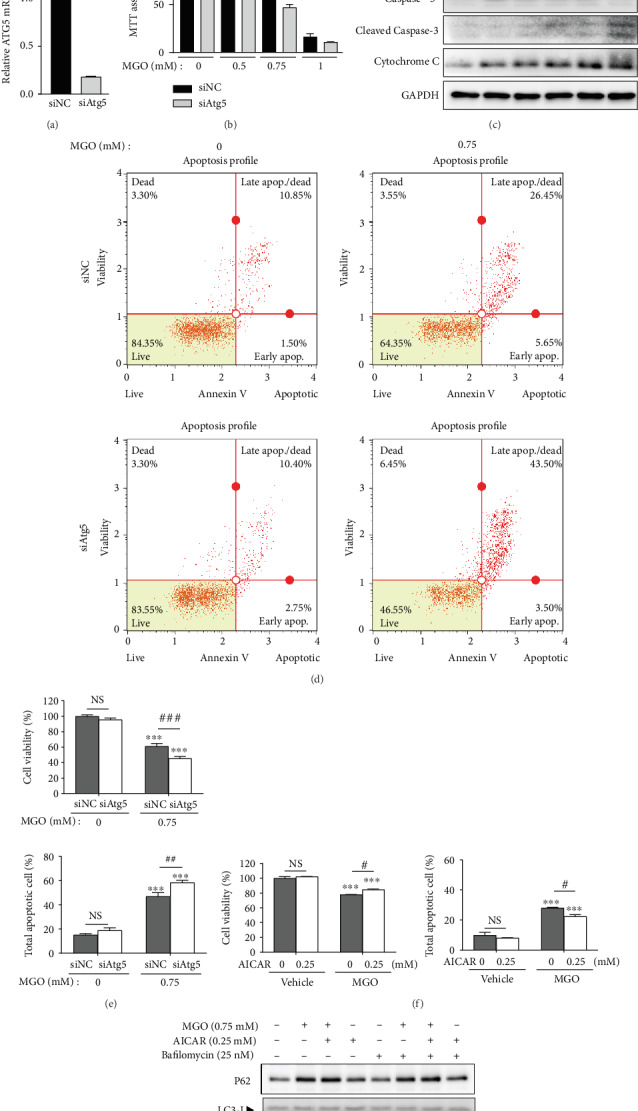
Autophagy exerts protective effects against MGO-induced apoptosis. (a) Relative *Atg5* mRNA levels of siNC and siAtg5 HK-2 cells. (b) Viability of transient siAtg5 cells treated with MGO (0–1 mM), as determined by MTT assays. (c) Comparison of apoptosis- and autophagy-related proteins in control cells and transient siAtg5-transfected cells treated with 0–0.75 mM of MGO for 24 h. (d) Representative images of flow cytometric analysis of apoptotic populations in control cells and transient siAtg5-transfected cells treated with 0.75 mM MGO for 24 h. (e) Percentages of live and apoptotic cells in the control and transient siAtg5-transfected groups treated with MGO. (f) Percentages of live and apoptotic cells cotreated with 0.75 mM MGO for 24 h and 0.25 mM AICAR for 8 h, as determined by flow cytometric analysis. (g) Expression of autophagy-related proteins in cells treated with MGO (24 h), AICAR (8 h), and baf (2 h). Values are mean ± standard deviation. ^∗∗^*p* < 0.01 and ^∗∗∗^*p* < 0.001 versus the control (0) group; ^#^*p* < 0.05, ^##^*p* < 0.01, and ^###^*p* < 0.001 versus the same concentration of MGO (*n* = 3 experiments).

Autophagy can induce autophagic cell death [28]. Therefore, we verified whether MGO-activated autophagy induces cell death or protects damaged cells. To confirm the role of autophagy in MGO-treated cells, we transfected them with Atg5 siRNA, a key subunit involved in autophagosome formation, and measured cell viability using MTT assays [29]. The viability of MGO-treated siAtg5 cells was found to be significantly lower than that of control cells (Figures 3(a) and 3(b)). Moreover, MGO increased the levels of apoptosis marker proteins, such as PARP, caspase-3, and cytochrome C, in siATG5 cells compared with their levels in siNC cells, suggesting that autophagy may protect cells from MGO-induced cell death (Figure 3(c)). Interestingly, MGO also increased Atg5 protein expression in both siAtg5 and siNC cells, suggesting that MGO contributes to the upregulation of autophagy-related proteins, such as p62 and Atg5. We next analyzed the percentage of apoptotic cells in siAtg5 cells treated with MGO and found that cell viability was decreased by approximately 15% compared with that of siNC cells. Additionally, the percentage of apoptotic cells among siAtg5 cells treated with MGO was approximately 12% higher than that among siNC cells (Figures 3(d) and 3(e)). We then investigated whether autophagy-activating drugs can reduce MGO-induced apoptosis. To this end, we treated cells with AICAR, an autophagy activator that acts via AMPK activation, along with MGO and found that AICAR significantly suppressed MGO-induced apoptosis (Figure 3(f)). Treatment with AICAR has been shown to further increase autophagy flux, and this suggests that increasing autophagy activity may reduce MGO-induced cell death (Figure 3(g)). Taken together, these results suggest that MGO-induced autophagy does not contribute to apoptosis, but rather protects against cell death.

### 3.4. MGO-Induced Autophagy Occurred via AMPK Independent of the mTOR Complex 1 (mTORC1) Pathway

**Figure 4:**
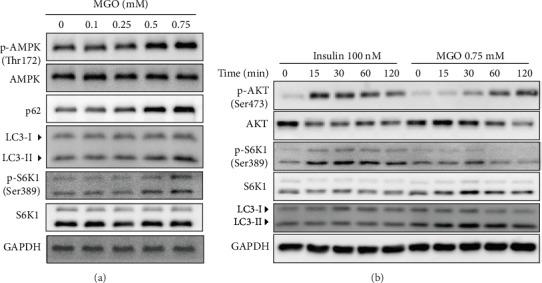
MGO upregulates autophagy independently of the mTORC pathway. (a) Western blot analysis of the effects of MGO treatment for 24 h on AMPK and autophagy-related proteins. (b) Time-dependent protein expression of MGO (0–120 min) or insulin treatment in serum-free media conditions (16 h).

MGO has been reported to act as an AMPK activator [26]. In this study, we confirmed that AMPK phosphorylation was increased in a concentration-dependent manner following MGO treatment (Figure 4(a), Figure S2). Interestingly, MGO also increased the expression of p-S6K1, an mTOR signaling protein. Since the mTOR pathway negatively regulates autophagy [30], it is necessary to confirm the effect of MGO on mTOR signaling. To this end, we treated HK-2 cells with insulin (mTOR signaling inducer) and MGO in serum-free conditions. Insulin treatment stimulated the phosphorylation of AKT at Ser473 and S6K1 at Ser389, which indicates the activation of mTORC1 signaling without altering the LC3 protein level. Alternatively, MGO treatment not only activated the phosphorylation of AKT and S6K1, but also increased LC3-II expression (Figure 4(b)). Taken together, these results suggest that MGO activated both autophagy and mTOR signaling and that autophagy is activated through an mTOR-independent pathway.

### 3.5. MGO Increased the Expression of Autophagy-Related Transcription Factors by Enhancing AMPK Activity

**Figure 5:**
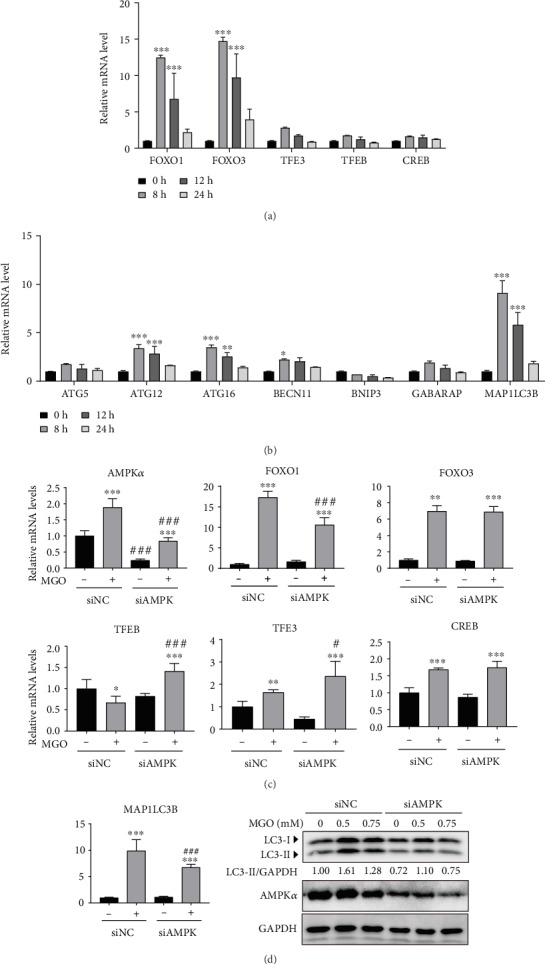
MGO upregulated the expression of autophagy-related transcription factors by enhancing AMPK activity. (a) Time-dependent mRNA levels of autophagy-related transcription factors induced by 0.75 mM of MGO. (b) mRNA levels of autophagy subunits modulated by transcription factors in HK-2 cells treated with MGO (0.75 mM). (c) Transcription factor mRNA levels of transient siAMPK-transfected cells compared with those of control cells treated with MGO for 8 h. (d) Expression of *MAP1LC3B* mRNA and LC3 protein level in siNC or siAMPK cells following treatment with MGO for 8 h (0.75 mM) or 24 h (0-0.75 mM), respectively. Values are mean ± standard deviation. ^∗^*p* < 0.05, ^∗∗^*p* < 0.01, and ^∗∗∗^*p* < 0.001 versus the control (0) group; ^#^*p* < 0.05 and ^###^*p* < 0.001 versus the same concentration of MGO (*n* = 3 experiments).

Another pathway for increasing autophagy may be transcription factor regulation. Activated AMPK regulates the forkhead box (FOXO) family of transcription factors [31]. Therefore, we analyzed mRNA levels of autophagy-related transcription factors, such as FOXO1, FOXO3, transcription factor EB (TFEB), transcription factor II E (TFE), and cAMP response element-binding protein [32]. FOXO1 and FOXO3 were significantly increased by MGO treatment (Figure 5(a)). Next, autophagy-related genes, such as Atg12, BNIP3, Beclin1, GABARAP, Atg16, and Atg7, which are transcriptionally regulated by FOXOs, were analyzed. MGO increased the expression of most autophagy genes, except BNIP3 (Figure 5(b)). To verify the influence of activated AMPK on transcription factor expression, we used transient siAMPK-transfected cells to confirm enhancement of transcription factor expression by AMPK activation. The results showed that FOXO1 expression, but not FOXO3 expression, was decreased by MGO treatment in siAMPK-transfected cells (Figure 5(c)). In addition, the expression of LC3B, which is regulated by FOXO1, was also significantly reduced by AMPK silencing (Figure 5(d)). These results indicated that MGO upregulated the mRNA expression of FOXO1 autophagy-related transcription factors by enhancing AMPK activity.

## 4. Discussion

Autophagy has been shown to inhibit or induce apoptosis in different stress environments; therefore, understanding the effects of autophagy and its mechanisms in different situations remains a major research focus [33]. In the absence of autophagy, MGO-induced apoptosis was significantly increased and treatment with an autophagy activator in addition to MGO significantly increased cell survival. Thus, autophagy in dicarbonyl stress appears to have a protective role in cells.

Although autophagy is controlled through various mechanisms, it is known that the mTOR pathway is a major regulatory pathway in autophagy [30]. Likewise, drugs known as mTOR inhibitors, namely, rapamycin and torin 1, have been reported to significantly increase autophagy [34, 35]. Moreover, AMPK is a master regulator of cellular energy homeostasis and has been shown to induce autophagy via inhibiting mTORC1 signaling through phosphorylation of raptor and TSC2, while also activating ULK1, which is associated with autophagy initiation [36]. Interestingly, our study demonstrated that MGO treatment activates S6K1, a protein downstream of mTORC1. Furthermore, in serum-free conditions, MGO not only induced autophagy but also increased AKT and S6K1 activity, similar to that observed following treatment with insulin. Normally, nutrient deficiency increases autophagy and suppresses the mTOR pathway. These results demonstrate that MGO increases autophagy and mTOR signaling, even under starvation conditions. Furthermore, MGO-induced autophagy was regulated via pathways independent of mTOR. However, both the induction of autophagy and mTOR signaling require further investigation.

AMPK also upregulates autophagy through the activation of specific transcription factors, including FOXOs, TFEB, TFE3, and CREB, which effectively upregulate autophagy by increasing the expression of autophagy-related genes [37–39]. Specifically, activated AMPK phosphorylates the Ser383 and Thr649 of FOXO1 to serve as a nuclear transcription factor. Alternatively, AKT phosphorylates the Thr24 and Ser256 of FOXO1, promoting the degradation of cytosolic FOXOs. Since MGO activated both AMPK and AKT, it is difficult to determine the effect of MGO on the transcriptional activity of FOXO1. However, according to a previous study, AMPK-mediated Ser22 phosphorylation of FOXO1 inhibits Thr24 phosphorylation by AKT, and subsequently promotes the degradation of FOXO1 [40]. Our data indicate that the activity of FOXO1 is directly regulated by AMPK in MGO-treated cells. Therefore, FOXO1 may be regulated by AMPK rather than AKT in MGO-treated HK-2 cells.

Specific limitations were noted in this study. First, all conclusions were drawn from *in vitro* analyses alone. Although, recently, diabetic nephropathy preclinical animal models have been reported, most of these mouse models only partially reflect the phenotype of diabetic nephropathy, such as hyperfiltration, mesangial expansion, and glomerular basement membrane thickening. Therefore, novel nephropathy models of complete diabetic kidney disease are needed to validate our findings [41]. Second, further studies are needed to determine whether increasing the activity of autophagy in diabetic nephropathy is effective in alleviating diabetic complications.

## 5. Conclusions

The data obtained through this study indicate that MGO induces both apoptosis and autophagy. MGO induced autophagy by inducing AMPK activity independently of mTOR signaling. Further, we found that autophagy reduced MGO-induced apoptosis through AMPK activation. MGO-activated AMPK increased FOXO1 expression, an autophagy-related transcription factor, resulting in an increased expression of autophagy-related genes. These findings are expected to be useful in carbonyl stress-related diseases, such as diabetic nephropathy treatment and prevention strategies.

## Figures and Tables

**Table 1:** Primer sequences.

DNA target	Sequence (5′ to 3′)	
*MAP1LC3B*	F	GATGTCCGACTTATTCGAGAGC
	R	TTGAGCTGTAAGCGCCTTCTA

*BECN11*	F	CCATGCAGGTGAGCTTCGT
	R	GAATCTGCGAGAGACACCATC

*FOXO3*	F	CGGACAAACGGCTCACTCT
	R	GGACCCGCATGAATCGACTAT

*TFE3*	F	CCGTGTTCGTGCTGTTGGA
	R	GCTCGTAGAAGCTGTCAGGAT

*TFEB*	F	ACCTGTCCGAGACCTATGGG
	R	CGTCCAGACGCATAATGTTGTC

*FOXO1*	F	TCGTCATAATCTGTCCCTACACA
	R	CGGCTTCGGCTCTTAGCAAA

*CREB*	F	TCAGCCGGGTACTACCATTC
	R	TCTCTTGCTGCTTCCCTGTT

*GABARAB*	F	AGAAGAGCATCCGTTCGAGAA
	R	CCAGGTCTCCTATCCGAGCTT

*ATG16L*	F	AACGCTGTGCAGTTCAGTCC
	R	AGCTGCTAAGAGGTAAGATCCA

*BNIP3*	F	CAGGGCTCCTGGGTAGAACT
	R	CTACTCCGTCCAGACTCATGC

*ACTIN*	F	CATGTACGTTGCTATCCAGGC
	R	CTCCTTAATGTCACGCACGAT

*AMPKα*	F	TTG AAA CCT GAA AAT GTC CTG CT
	R	GGT GAG CCA CAA CTT GTT CTT

*ATG5*	F	TGTGCTTCGAGATGTGTGGTT
	R	GTCAAATAGCTGACTCTTGGCAA

*ATG12*	F	TAGAGCAACACGAACCATCC
	R	CACTGCCAAAACACTCATAGAGA

*p62/SQSTM-1*	F	GACTACGACTTGTGTAGCCTC
	R	AGTGTCCGTGTTTCACCTTCC

## Data Availability

The data used to support the findings of this study are available from the corresponding authors upon request.

## Supplementary Materials

Supplementary Materials Figure S1: cellular viability of MGO-treated SV40MES13 cells. SV40MES13 cells were treated with various concentrations of MGO (0-0.75 mM) for 24 h, and cell viability was measured using the MTT assay. Figure S2: protein levels of autophagy-related genes in MGO-treated SV40MES13 cells. SV40MES13 cells were seeded in a 6-well plate (2 × 10^5^ cells per well). On the next day, the cells were treated with 0–0.25 mM of MGO for 24 h. The cells were harvested and analyzed by immunoblotting.
