# Supplementary material for: Autophagy Functions to Prevent Methylglyoxal-Induced Apoptosis in HK-2 Cells
Source: Oxid Med Cell Longev. 2020 Jun 4;2020:8340695. doi: 10.1155/2020/8340695 (PMC7292969; doi:10.1155/2020/8340695)
Supplement: Supplementary Materials — Figure S1: cellular viability of MGO-treated SV40MES13 cells. SV40MES13 cells were treated with various concentrations of MGO (0-0.75 mM) for 24 h, and cell viability was measured using the MTT assay. Figure S2: protein levels of autophagy-related genes in MGO-treated SV40MES13 cells. SV40MES13 cells were seeded in a 6-well plate (2 × 10^5^ cells per well). On the next day, the cells were treated with 0–0.25 mM of MGO for 24 h. The cells were harvested and analyzed by immunoblotting. [file 8340695.f1.docx]

**Supplementary materials**


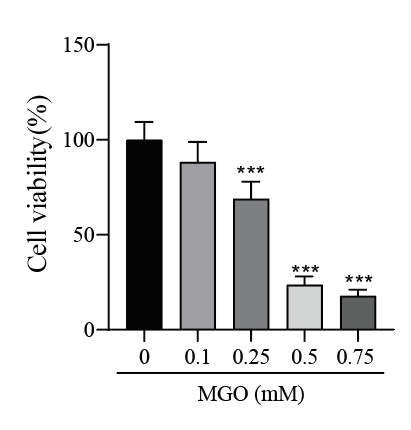


Figure S1 – Cellular viability of MGO-treated SV40Mes13 cells. SV40Mes cells were treated with various concentration of MGO (0-0.75 mM) for 24 h and cell viability was measured using the MTT assay.


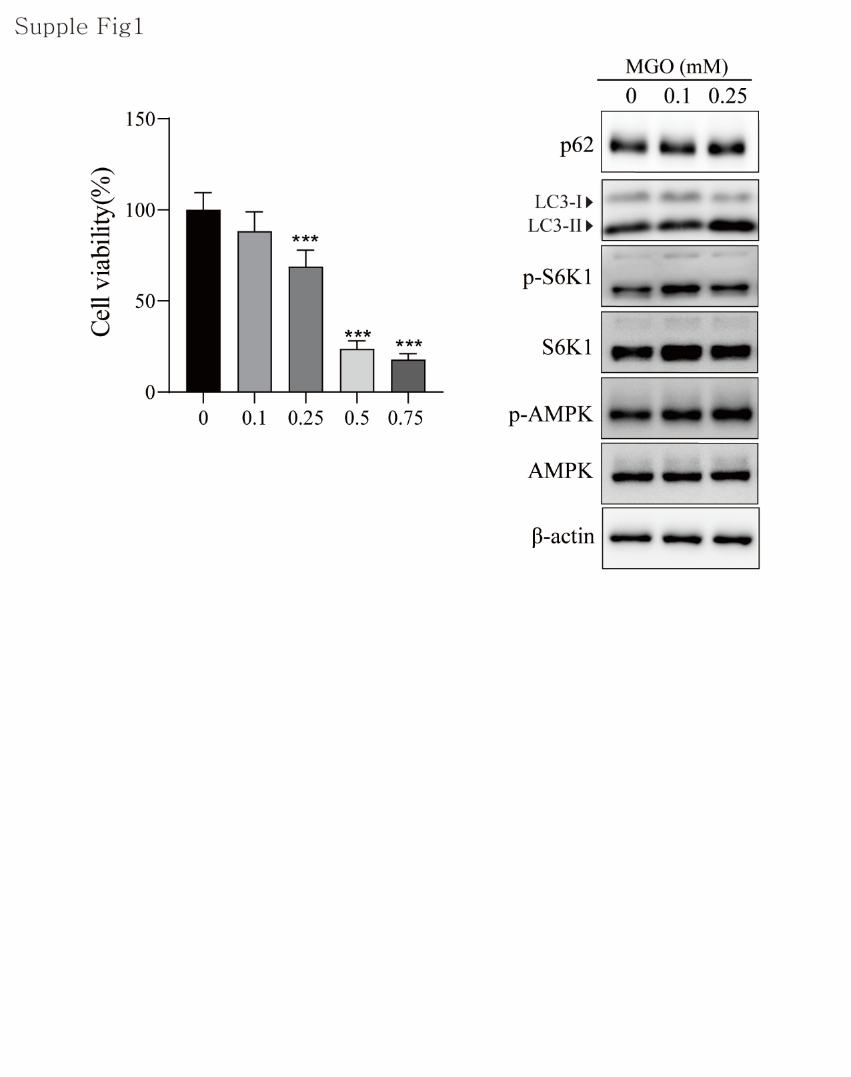


Figure S2 – Protein levels of autophagy-related genes in MGO-treated SV40Mes cells. SV40Mes cells were seeded in a 6-well plate (2 ×10^5^ cells per well). Next day, cells were treated with 0–0.25 mM of MGO for 24 h. The cells were harvested and analyzed by immunoblotting.
